# Review of *Feminist Bioethics At the Center, On the Margins*, edited by Jackie Leach Scully, Laurel E. Baldwin-Ragaven, Petya Fitzpatrick

**DOI:** 10.1186/1747-5341-5-18

**Published:** 2010-12-06

**Authors:** Maureen Sander-Staudt

**Affiliations:** 1 Division of Humanities, Arts, and Cultural Studies, Arizona State University, PO Box 37100, Phoenix, AZ 85069-7100, USA

## Abstract

The anthology, *Feminist Bioethics*, edited by Jackie Leach Scully, Laurel E. Baldwin-Ragaven, and Petya Fitzpatrick, examines how feminist bioethics theoretically and methodologically challenges mainstream bioethics, and whether these approaches are useful for exploring difference in other contexts. It offers critical conceptual analyses of "autonomy", "universality", and "trust", and covers topics such as testing for hereditary cancer, prenatal selection for sexual orientation, midwifery, public health, disability, Indigenous research reform in Australia, and China's one child policy.

## Review

*"Supposing truth is a woman--what then? Is there not ground for suspecting that all philosophers, in so far as they have been dogmatists, have been very inexpert about women?" ~ Friedrich Nietzsche *[1]

Nietszsche can hardly have been considered a feminist in his time, much less a bioethicist, but his 1886 question relating truth and sex-based experience has relevance for bioethical studies today, as demonstrated by the new anthology, *Feminist Bioethics*, edited by Jackie Leach Scully, Laurel Baldwin-Ragaven, and Petya Fitzpatrick. This collection features fourteen essays by authors committed to revealing bioethical insights putting questions of sex and gender at the fore, and taking women's experiences seriously. As the editors put it, "feminist bioethics starts from the premise that dominant ways of doing bioethics are *fundamentally *gendered and that they thus contribute to culturally inscribed oppressive practices" (3, original emphasis). According to the editors, mainstream bioethics encourages oppression in two ways--first, by featuring subject matter that reflects masculine experience and priorities, and second, by developing ontological and epistemological foundations that privilege ways of knowing that are masculine, devaluing what is culturally coded as feminine. With commentary from the editors throughout, this book details how mainstream bioethics benefits from the inclusion of feminist analyses, and is of interest to anyone who has an interest in bioethics and diversity.

The book is divided into four sections, tracing how feminist bioethics theoretically and methodologically challenges mainstream bioethics, and how these approaches may or may not be useful for exploring difference in other contexts. It begins with a triad of essays providing an historical overview of feminist bioethics. In the lead essay, Anne Donchin reviews the accomplishments and potential areas of future scholarship of the International Network for Feminist Approaches to Bioethics (FAB, founded in 1992), and its new journal, the *International Journal of Feminist Approaches to Bioethics (IJFAB*, published first in 2008). Christoph Rehman-Sutter similarly examines three editions of *The Encyclopedia of Bioethics*, tracing over two decades of change in mainstream bioethics, attributable to, but not often accredited to feminism-- such as the treatment of the topic of prenatal diagnosis. Richard Twine deploys a multi-faceted feminist framework to reveal omissions and areas of de-emphasis in feminist bioethics, developing a critical bioethics consisting of interdisciplinarity, self-reflexivity, and avoidance of uncritical complicity. He argues for widening the meaning of "bio" in bioethics to include environmental issues, such that bioethics would become a movement for environmental justice. Twine is hopeful that interdisciplinary, coalitionary endeavors can contest the domains considered bioethically relevant, and counter the alienation of the body and emotion in bioethics.

The second section of *Feminist Bioethics *critically engages the philosophical foundations of dominant bioethics. The authors in this section set out feminist objections to some of the standard conceptual underpinnings of mainstream bioethics, including "autonomy", "universality", and "trust". Launching critiques of the common understandings of these concepts, Catriona Mackenzie and Mary Rawlinson use feminist philosophies to revise the concept of "autonomy" understood as "maximal choice", and also "universality", which it is contended hides masculine biases behind a façade of neutral equality. MacKenzie construes autonomy as socially constituted and ideally attentive to the effects of power relations on individual choices, opportunities, and capacities. Rawlinson details how the *Universal Declaration on Bioethics and Human Rights*, adopted by the General Conference of UNESCO in 2005, sets well-meaning universal standards that nonetheless obscure historical links between abstract rights discourses and practical inequalities. Similarly, Jessica Prata Miller explores differences between feminist and mainstream conceptualizations of trust as a relational ideal. According to Miller, the central feminist lessons about trust are that "trust is not to be trusted", and that bioethicists ought more often focus on cases where trust is not warranted or is non-paradigmatic (100; 103).

The third section of *Feminist Bioethics *contains some of its most innovative work, applying feminist methodology to questions of how research is conducted, arguments are framed, and methodological conflicts are mediated in bioethics. As the editors point out, feminist methodologies often are difficult to discern as distinctly feminist. What makes a methodology feminist is not simply that it addresses women's issues, but that it highlights personal and political aspects of experience, attends to power relations, and spurs social and political change. All of these methodological aspects are present in Lori D'Agincourt-Canning's essay "Bodies, connectedness, and knowledge: A connected approach to hereditary cancer genetics". Exposing some of the potential harms and benefits of new genetic diagnostic technologies, D'Agincourt-Canning examines how individuals from families with hereditary cancer experience and construct knowledge about breast and ovarian cancer. Such knowledge is not individualistic or purely cerebral, but is built on empathy with family members who have experienced cancer, and from embodied experiences with cancer, both first and second hand. D'Agincourt-Canning observes that in this context the choice to be tested for a cancer related gene is not a one time decision that affects an individual alone, but one that has inter-generational implications. Stressing the relational implications of genetic testing as well as feminist tensions with ideals of autonomy, she surmises that being a good family member who is responsible with genetic information may involve relinquishing choices that in other settings could be viewed as an unwarranted constriction of autonomy.

The three authors who follow D'Agincourt-Canning in this section similarly pose challenges to liberalism and exemplify how the idea that "the personal is political" can serve as a methodology in feminist bioethics. In "Stories of innocence and experience: Bodily narrative and rape", Fiona Utley uses an embodied approach to reconstruct the aftermath of rape, challenging the tendency to view rape victims as suffering from post-traumatic stress disorder. Within the framework of a narrative phenomenology, Utley recounts that "traumatic memory is not narrative, but experience that reoccurs" [2]. Experienced by a mind-body subject, traumatic memory of rape is intimately tied to the body in ways that destroy a victim's sense of safety, replace cognitive and emotional capacities with feelings of numbness, and erode the ability to form a "rational life plan", so central to liberal philosophies like that of John Rawls. Ultimately, Utley resists against absorbing the aftermath of rape into cultural narratives of illness because this burdens victims with a private responsibility for recovery.

Other authors in this section attend to the use of technology at the stages of pregnancy and birth. Looking at the ethics of genetic testing at pre-natal stages of human life, Janice Mclaughlin argues against parental liberty to select the sexual orientation of unborn children, because the choice to select against homosexuality conflicts with the ideal of providing gays and lesbians with equal social space, and supports discriminatory cultural norms. She writes, "to preselect to avoid the future harm that being gay is assumed to involve helps to construct the world as a place where that harm takes place" (185). With similar discomfort over the increasingly unbridled use of technology in the birthing process, Al-Yasha Ilhaam and Ina May Gaskin argue against viewing of birth as fetus-centered and fundamentally in need of medical intervention. In their essay "Toward a methodology for technocratic transformation" they endorse a holistic model that construes childbirth not as a perilous malady, but as a generally non-threatening life occurrence, when facilitated by good maternal care. They caution that a rise in cesarean sections and maternal death in mainstream obstetric practices indicates that doctors' hands on birthing skills may be atrophying in favor of technological reactionary solutions.

The book concludes with four essays exploring how feminist bioethics may or may not serve to forward the interest of other marginalized groups. In "The difference that difference makes: Public health and the complexities of racial and ethnic differences", Ruth Groenhout seeks to "approach disparities without denying them", using a care based theory in place of the principle based theories favored by mainstream bioethics. She notes that although the National Institute of Health requires the inclusion of both women and minorities in medical studies, it is common for studies to ignore both groups. Because "difference" is understood hierarchically in the West, as being inferior from the presumed norm, it is unlikely that merely collecting data based on racial, ethnic, or gender differences will result in better treatment for the members of such groups. More troubling, research that takes difference into account can be used in ways harmful to these groups, by entrenching disease etiologies as unfixable and genetically determined--as just "how those folks are" (228). Groenhout recommends new research protocols that include minority populations on the ground level of research planning, and that avoid paternalistically serving the interests of the powerful at the expense of the vulnerable. Demonstrating such a case of vulnerability, Jennifer Baker, Terry Dunbar, and Margaret Scrimgeor explore past abuses in Australian Indigenous research, and the potential of feminist bioethics to lead research reform in this area. They note that viewing Indigenous people as "objects" of inquiry is exploitative and unlikely to lead to sustainable or positive change. Akin to Groenhout, they recommend a feminist bioethical framework capable of disclosing intersecting oppressions of gender, race, and globalization, and Indigenous community involvement at all stages of the research process.

*Feminist Bioethics *ends on high notes with essays from Jing-Bao Nie and Mary Mahowald. While the former scrutinizes the morality of China's population policy of *Yitai Hua *(one-child), aimed at producing "fewer but healthier births", the latter develops a feminist standpoint on disability. Arguing for the necessity of a woman-centered population policy in China, Nie chastises both feminist and mainstream bioethics for ignoring a policy that affects more than 1/5 of all women in the world, and has resulted in an estimated "40 million missing girls" (266). Acknowledging that the policy has helped to improve living standards for many Chinese women, Nie laments how women's bodies have borne the burden of abortion and contraception associated with the policy, and how it also has helped to transform China into a "hard-edged, competitive" society of "consumerist singletons exist[ing] in a larger cultural sea of peasant suffering and female sacrifice"[3]. Nie calls for feminist bioethicists to amplify the voices of Chinese women, and cultivate a language rooted in Daoism or Confucianism for underprivileged and marginalized people.

In the final essay of *Feminist Bioethics*, Mary Mahowald develops a standpoint theory from the perspective of women with disabilities, using it to defend feminist egalitarianism and atypical meanings of "disability". Arguing for a broader "flourishing" based standard of justice over a more narrow "function" based norm, Mahowald affirms the right of those who are disabled to not only function as others do, but to flourish as unique individuals with different mixes of abilities and disabilities. She notes that even those who cannot "normally function" can flourish as themselves, and that a more ambitious ideal of flourishing means attending the needs of all individuals as individuals. Twisting the typical understanding of "disability" as a condition that reduces the capabilities of some non-dominant members of society, Mahowald invites consideration to how "disability" could also apply to the condition of reduced capabilities based on social arrangements, such as poverty, or minority status (280). Although Mahowald endorses the goal of flourishing as a social ideal, she stipulates that when flourishing cannot be met, function-based standards should be met for all, before the flourishing of only some.

As a whole, *Feminist Bioethics *is an important new addition to bioethical literature, demonstrating that feminism has global and interdisciplinary relevance to a wide range of bioethical topics. In addition to shifting the focus and meaning of key concepts in mainstream bioethics, *Feminist Bioethics *opens up exciting new modes of inquiry in the field, and exposes how existing bioethical methodologies owe an unacknowledged debt to feminism. Although the book does not include a full review of feminism of the sort that may be useful to readers new to feminist theory, it is commendable for not limiting itself to care theory, and for resisting the common tendency (largely of nonfeminists) to reduce all of feminist ethics to an ethics of care. It is also commendable that the book strives to include diverse perspectives, and attends to global concerns beyond dominant Western interests. On this topic the editors acknowledge with regret that there are many voices missing from the book yet to be heard, but rightly project that in the future feminist bioethics will be even more global and diverse. As it is, *Feminist Bioethics *is a book that unites academic and activist communities, uses many concrete examples adaptable to classroom use, and demonstrates the current and future relevance of sex/gender issues to biological life studies and beyond.

## List of Abbreviations

UNESCO: United Nations Educational, Scientific, and Cultural Organization

## About the Author

Maureen Sander-Staudt is an Assistant Professor at Arizona State University-West. She specializes in feminist ethics, with interests in care ethics, bioethics, and social and political philosophy. She has published in journals such as *Hypatia *and *The Journal of Social Philosophy*. She is currently co-editing two anthologies, *Philosophical Inquiry into Pregnancy, Childbirth, and Motherhood*, forthcoming with Routledge, as well as *Applying Care Ethics to Business Ethics*, with Springer Press.

## Competing interests

The author declares that they have no competing interests.
